# Transmission of antibiotic resistance at the wildlife-livestock interface

**DOI:** 10.1038/s42003-022-03520-8

**Published:** 2022-06-15

**Authors:** Shinyoung Lee, Peixin Fan, Ting Liu, Anni Yang, Raoul K. Boughton, Kim M. Pepin, Ryan S. Miller, Kwangcheol Casey Jeong

**Affiliations:** 1 Emerging Pathogens Institute, University of Florida, Gainesville, FL 32611 USA; 2 Department of Animal Sciences, University of Florida, Gainesville, FL 32611 USA; 3 Department of Fish, Wildlife, and Conservation Biology, Colorado State University, Fort Collins, CO 80523 USA; 4 National Wildlife Research Center, United States Department of Agriculture, Animal and Plant Health Inspection Service, Wildlife Services, 4101 Laporte Ave., Fort Collins, CO 80521 USA; 5 Range Cattle Research and Education Center, Wildlife Ecology and Conservation, University of Florida, Ona, FL 33865 USA; 6 Center for Epidemiology and Animal Health, United States Department of Agriculture, Animal and Plant Health Inspection Service, Veterinary Services, 2150 Center Dr., Fort Collins, CO 80523 USA

**Keywords:** Environmental microbiology, Antimicrobials

## Abstract

Antibiotic-resistant microorganisms (ARMs) are widespread in natural environments, animals (wildlife and livestock), and humans, which has reduced our capacity to control life threatening infectious disease. Yet, little is known about their transmission pathways, especially at the wildlife-livestock interface. This study investigated the potential transmission of ARMs and antibiotic resistance genes (ARGs) between cattle and wildlife by comparing gut microbiota and ARG profiles of feral swine (*Sus scrofa*), coyotes (*Canis latrans*), cattle (*Bos taurus*), and environmental microbiota. Unexpectedly, wild animals harbored more abundant ARMs and ARGs compared to grazing cattle. Gut microbiota of cattle was significantly more similar to that of feral swine captured within the cattle grazing area where the home range of both species overlapped substantially. In addition, ARMs against medically important antibiotics were more prevalent in wildlife than grazing cattle, suggesting that wildlife could be a source of ARMs colonization in livestock.

## Abstract (web summary)

Analysis of microbiome data from feral swine, coyotes, domesticated cattle, and the surrounding environment reveals that wild animals harbor more abundant antibiotic-resistant organisms than livestock, and might act as a source of antibiotic-resistant microbes in outbreaks.

## Introduction

Antibiotics play a critical role in preventing and treating diseases in humans and animals. However, overuse of antibiotics has caused the emergence of antibiotic-resistant microorganisms (ARMs), one of the biggest global challenges threatening both public and animal health^1^. To mitigate the prevalence of ARMs especially in animals, many countries have banned clinically important antibiotics for non-therapeutic use in livestock, and recommended using veterinary-prescribed antibiotics for therapeutic purposes^2^. Significant efforts, such as antibiotic stewardship for selection, dosage, and duration of treatment, are imposed to reduce the prevalence of multi-drug resistant bacteria that are still prevalent in food-producing animals^3–8^.

Living in the same space can increase the similarity of bacterial community composition among individuals due to increased sharing of resources among different hosts^9–11^. For example, people who live with pets have more similar microbiota as their pets, indicating microbiota transmission between pet owners and their pets^12,13^. Spouses living together have similar gut microbiota profiles than sibling pairs living apart, and the length of cohabitation is positively correlated with the similarity of microbiota structure^14^. Tung et al.^15^ found that rates of interaction among wild baboons within cohabitating groups can explain variation in the gut microbiome, suggesting that close social interactions among hosts influence gut microbiota composition. Similarly, the bacterial community of bird’s bodies is associated with nest microbiota that shows microbiota transmission between birds and nests^16^. All these studies indicate that the interactions between hosts and surrounding external environments are an important extrinsic factor shaping gut microbiota^17^.

Cattle on cow/calf operations in the United States graze on pastures that often overlap areas that are shared by a variety of wildlife species. This can allow for cross-species transmission of microbes through either direct (nose-to-nose contact) or indirect (via shared food or water sources) mechanisms, depending on the biosecurity practices, with the potential of transmitting pathogens^18,19^. Previously, we reported a high prevalence of ARMs in cattle raised without antibiotic use^4–6^. During the first year of life, over 92% of cattle were colonized by ARMs at least once^5^. Also, ARMs isolated from cattle raised without antibiotic treatment carried multiple drug resistance genes^6^. These results suggest that cattle may be acquiring ARMs through other mechanisms than selection by antibiotic exposure. As wildlife are reservoirs for many livestock and zoonotic diseases including rabies, bovine tuberculosis, pseudorabies, and brucellosis^20^, we hypothesized that cattle sharing space with wildlife may acquire their ARMs. In this study, we aimed to identify potential transmission pathways of ARMs at the interface of wildlife and livestock by comparing the microbiota composition and antibiotic resistance genes (ARGs) between beef cattle and wildlife according to their home ranges.

## Results

### High prevalence of ARMs in wildlife

**Fig. 1:**
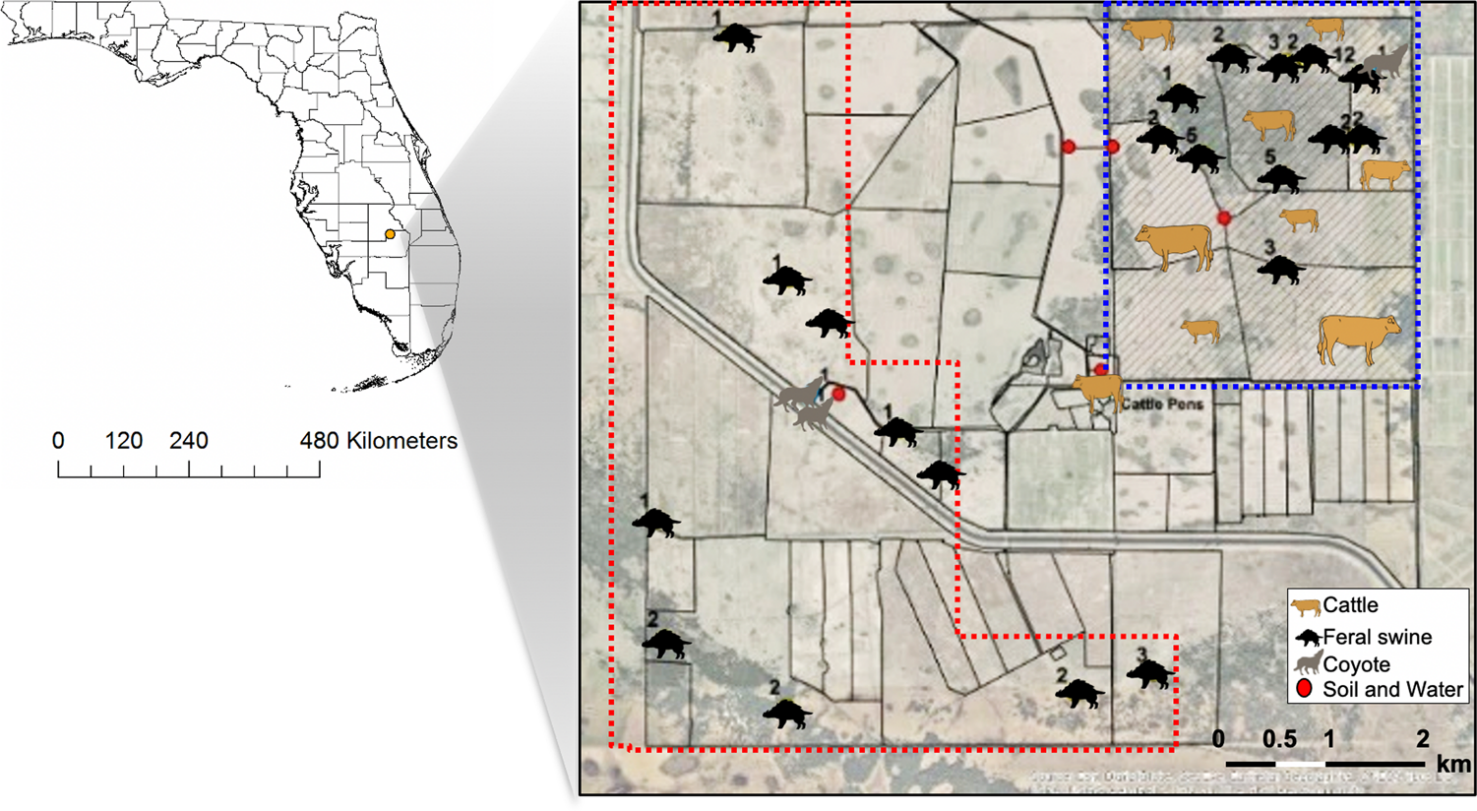
Location of samples. The samples used in this study were collected from South Florida. Feral swine were divided into ‘FWCGA’ (dashed blue line) and ‘FOCGA’ (dashed red line) groups based on where samples were collected. Labels represent the number of animals collected at each spot. The county-level map of Florida was acquired through ArcGIS online, created by the EsnTrainingSvc, and the map was visualized using ArcMap 10.5.

**Fig. 2:**
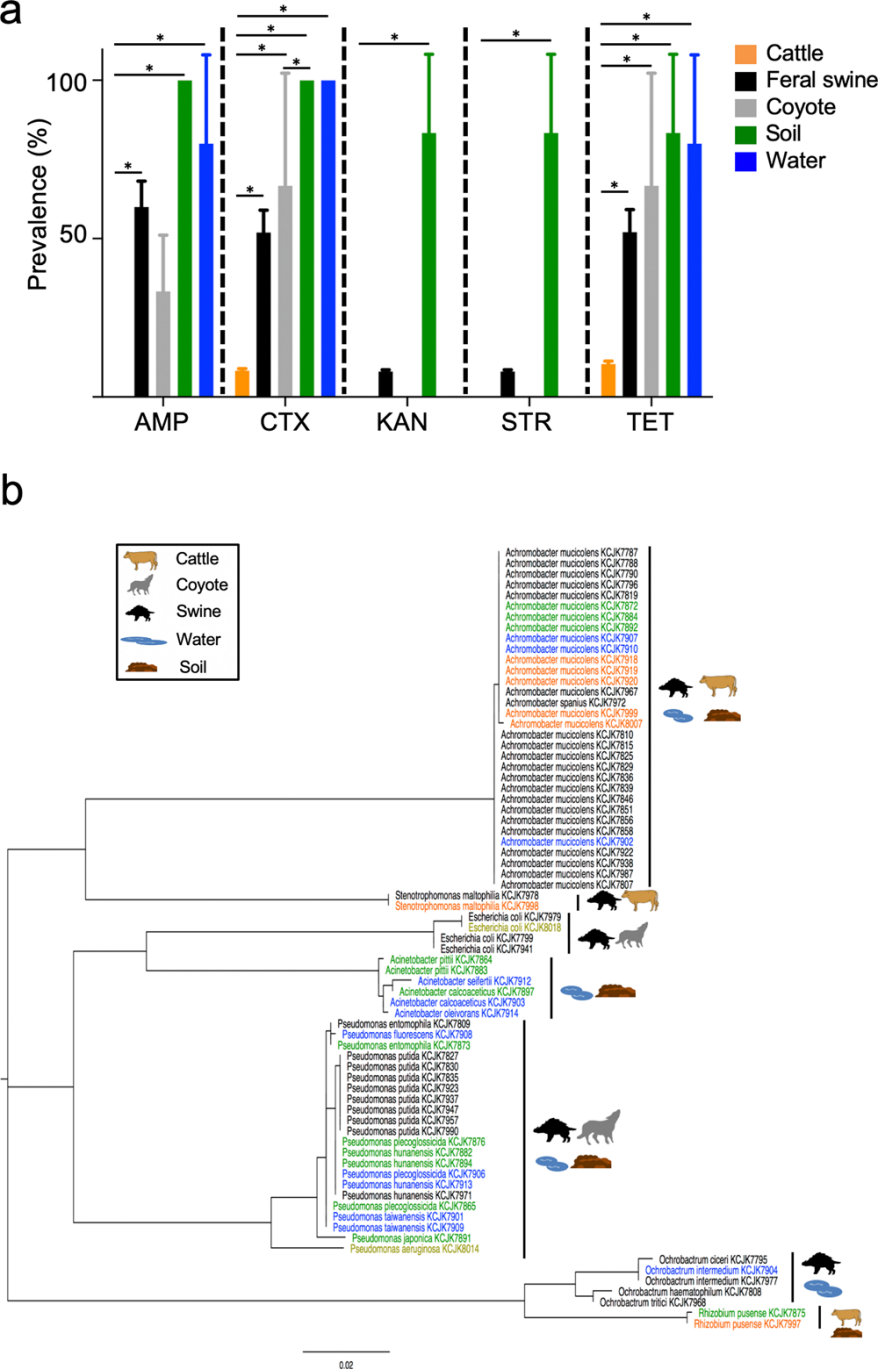
Prevalence of antibiotic-resistant microorganisms (ARMs) at the wildlife-livestock interface. **a** The prevalence of ARMs by type of antibiotic (AMP ampicillin, CTX cefotaxime, KAN kanamycin, STR streptomycin, TET tetracycline). The error bars indicate 95% confidence intervals. The prevalence of ARMs among different antibiotics were also compared using a Chi-Square test as follows: cefotaxime vs. kanamycin in feral swine (*P* < 0.0001), cefotaxime vs. streptomycin in feral swine (*P* < 0.0001), tetracycline vs. kanamycin in feral swine (*P* < 0.0001), tetracycline vs. streptomycin in feral swine (*P* < 0.0001), cefotaxime vs. kanamycin in cattle (*P* < 0.0001), cefotaxime vs. streptomycin in cattle (*P* = 0.041), tetracycline vs. kanamycin in cattle (*P* = 0.041), tetracycline vs. streptomycin in feral swine (*P* = 0.021). Asterisks indicate a statistical difference (*P* < 0.05) of each category comparison by Chi-Square test. **b** A phylogenetic tree of 16 S rRNA sequences from cefotaxime resistant bacteria estimated by maximum likelihood. Different colors indicate source of the isolate (black: feral swine, orange: cattle, olive: coyote, green: soil, blue: water).

To understand the prevalence of ARMs at the wildlife-livestock interface (Fig. 1), we selected ARMs with ampicillin, cefotaxime, kanamycin, streptomycin, or tetracycline antibiotics from samples collected (Fig. 2a and Supplementary Data 1). Tetracycline and cefotaxime-resistant bacteria were isolated from all sources of samples, whereas kanamycin and streptomycin-resistant bacteria were only found in soil and feral swine samples. The prevalence of cefotaxime-resistant bacteria (CRB) ranged from 8.3% (cattle) to 100% (water and soil). Interestingly, the prevalence of CRB in feral swine (51.92%, *P* < 0.0001) was significantly higher than in cattle. These CRB data were surprising because a third-generation cephalosporin antibiotic cefotaxime has not been allowed for prophylactic use in food-producing animals due to its importance for medical use in humans^21^, and it is only rarely used for animal disease treatment^22^.

We further speciated CRB isolates from wildlife and livestock samples to understand the diversity of CRB by 16 S rRNA gene sequencing. *Achromobacter* spp. were the most predominant CRB followed by *Pseudomonas* spp., *Acinetobacter* spp., *Ochrobactrum* spp., and *Escherichia coli* (Fig. 2b). The samples from all sources contained *Achromobacter* spp. except coyotes. *Pseudomonas* spp. were isolated from feral swine, coyotes, water, and soil, but not cattle. In addition, the cefotaxime-resistant opportunistic pathogen, *Stenotrophomonas maltophilia*^23^ was isolated from feral swine and cattle, and a human pathogen, *Rhizobium pusense*^24^ was in cattle and soil (Fig. 2b). Taken together, the same CRB are predominant among the samples, suggesting potential transmission of ARMs at the wildlife-livestock interface.

### Distinct microbiota structure of cattle, wildlife, and the environmental samples

In addition to the 16 S rRNA gene sequencing of culturable bacteria (Fig. 2), in order to understand the potential transmission of CRB at the wildlife-livestock interface, we analyzed microbiota composition and structure by using 16 S rRNA gene sequencing of 113 samples from cattle (n = 47), feral swine (n = 52), coyotes (n = 3), soil (n = 6), and water (n = 5). In total, 13,360,504 raw sequencing reads were obtained, and 6,034,513 reads contained Operational Taxonomic Units (OTUs) clustered into 162,391 OTUs. The average number of observed OTUs in each sample source was 1835 in cattle, 986 in feral swine, 492 in coyote, 3,080 in soil, and 1,370 in water samples, respectively (Supplementary Data 2).

**Fig. 3:**
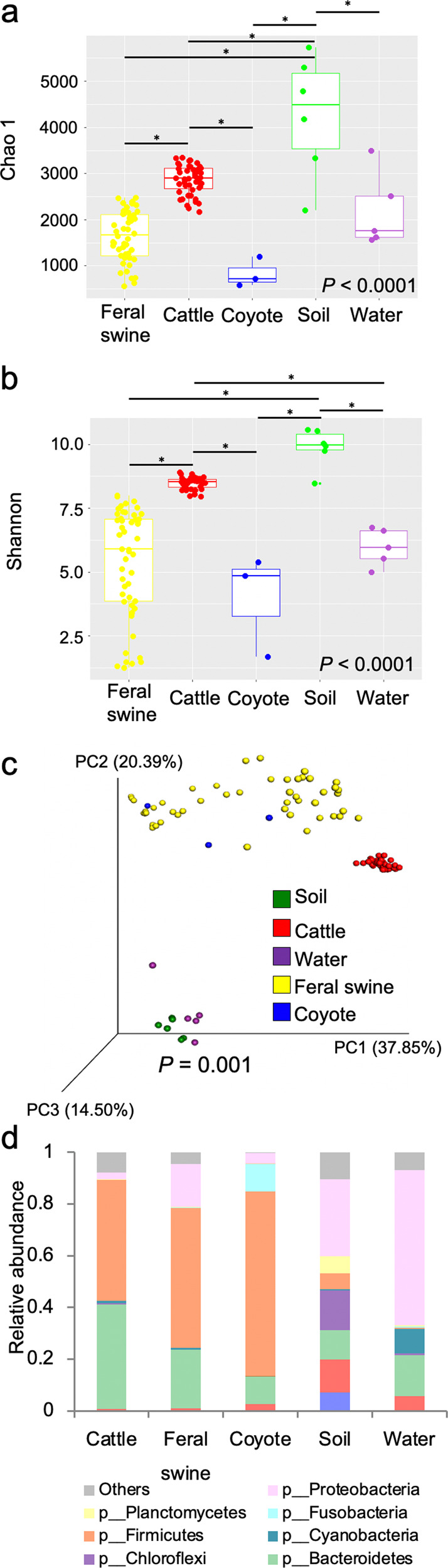
Microbiota structure in animal feces and environmental samples. **a** Bacterial richness of cattle, wildlife, and environmental samples. **b** Bacterial diversity of cattle, wildlife, and environmental samples. Box plots display the median (center line), 25th and 75th percentiles (box) and 5th and 95th percentiles (whiskers) for each individual. Asterisks indicate a statistical difference (*P* < 0.05) of each category comparison. **c** The principal coordinate analysis (PCoA) plot based on weighted UniFrac distances. PCoA plots demonstrated distinct microbiota structure of cattle, wildlife, and environmental samples (*P* = 0.001). **d** The relative abundance of bacteria in cattle, wildlife, and environmental samples at the phylum taxonomic level.

Overall, alpha-diversity measured by bacterial richness (Chao 1, *P* < 0.0001) and diversity (Shannon, *P* < 0.0001) were significantly different among sources. Soil samples showed the highest bacterial richness and diversity, followed by cattle, water, feral swine, and coyote samples (Fig. 3a, b, and Supplementary Data 1). Within animal groups, bacterial richness and diversity were significantly higher in cattle than in feral swine and coyotes (*P* < 0.0001, Fig. 3a, b). Beta-diversity measured by weighted UniFrac distances accounting for dissimilarity in both presence and abundance of bacteria was significantly different among sources (*P* = 0.001). As shown in the principal coordinate analysis (PCoA) plot, PC1, PC2, and PC3 explain 37.85%, 20.39%, and 14.50% of the variation, respectively in microbiota composition among sources (Fig. 3c, and Supplementary Data 1*P* = 0.001). Environmental samples (soil and water) and cattle microbiota were clustered closely based on their sample types, while microbiota of feral swine and coyotes were loosely clustered, indicating feral swine and coyotes have a more heterogeneous microbiota structure than cattle (Fig. 3c). While the relative abundances of bacteria were different in each animal group, Firmicutes and Bacteroidetes were the most prevalent phyla, accounting for 87%, 77%, and 82% in cattle, feral swine, and coyotes, respectively (Fig. 3d). Proteobacteria and Fusobacteria were another major phyla found in feral swine and coyotes, respectively. Environmental samples showed diverse bacterial composition compared to animal groups, and Proteobacteria was the most abundant phylum in both soil and water. Unlike gut microbiota of animals, the proportion of Firmicutes was low in environmental samples. Soil samples showed a higher proportion of Acidobacteria, Actinobacteria, Chloroflexi, and Planctomycetes than other samples, and water samples contained more Cyanobacteria (Fig. 3d).

Taken together, samples collected from different sources had distinct microbiome composition and structure.

### Cohabitation of feral swine and cattle in the same home range affects microbiota composition

**Fig. 4:**
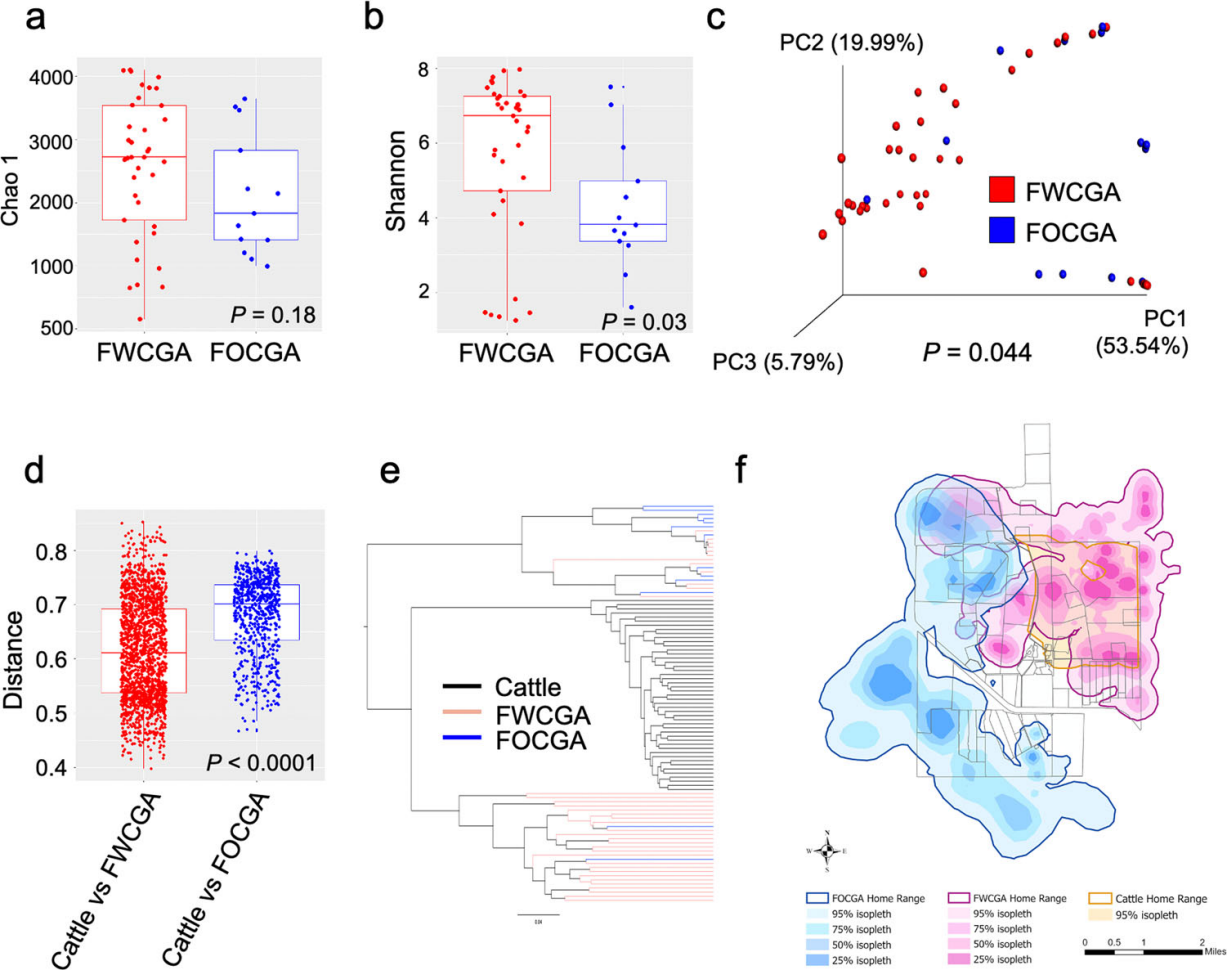
Comparison of the microbiota structure between cattle and feral swine groups. Bacterial richness (**a**) and diversity (**b**) of FWCGA and FOCGA. **c** The PCoA plot derived from weighted UniFrac distances. **d** The boxplot represents a comparison of weighted UniFrac distances between cattle vs. FWCGA and cattle vs. FOCGA, respectively. **e** Unweighted Pair Group Method with Arithmetic (UPGMA) tree based on weighted UniFrac distances shows the relatedness of microbiota between individual hosts. **f** Utilization distributions (UDs) and the 95% isopleth of the UDs for cattle (*n* = 11), FWCGA (*n* = 22), and FOCGA (*n* = 5) from April to May in 2017. Box plots display the median (center line), 25th and 75th percentiles (box) and 5th and 95th percentiles (whiskers) for each individual.

To further understand the heterogeneous microbiota structure of feral swine, we tested the hypothesis that geographical location is an important extrinsic factor shaping their gut microbiota. We divided feral swine into two groups based on their sampling location: feral swine caught within cattle grazing areas (FWCGA) and feral swine caught outside cattle grazing areas (FOCGA) (Supplementary Data 2 and Fig. 1). Bacterial richness (Chao 1, *P* = 0.18) was not significantly different (Fig. 4a and Supplementary Data 1), but diversity (Shannon, *P* = 0.03) was significantly different between FWCGA and FOCGA (Fig. 4b and Supplementary Data 1) which is consistent with our hypothesis. Beta-diversity shown in the PCoA plot measured by weighted UniFrac distances showed significant dissimilarity between the two groups (Fig. 4c, *P* = 0.044) as well. To investigate whether the age of feral swine affected the microbiota composition, feral swine were divided into juvenile (3–12 months) and adults (≥12 months) groups. The juvenile group had significantly higher bacteria diversity (*P* = 0.037) and tendency of higher richness (*P* = 0.063) compared to the adult group (Supplementary Fig. 1a, b). In addition, significant differences were observed in the overall microbiota structure between juvenile and adult swine based on the PCoA plot (Supplementary Fig. 1c, *P* = 0.012), suggesting that geographic location and animal factors influenced the composition of the microbiota. However, all juvenile swine were caught within cattle grazing areas while adult swine were caught inside and outside cattle grazing areas, we further compared the microbiota composition of juvenile and adult feral swine of FWCGA and FOCGA. Alpha diversity of gut microbiota was similar between juvenile and adult in FWCGA (*P* = 0.068 and *P* = 0.056) (Supplementary Fig. 1d–e), but it was significantly different between juvenile and adult in FOCGA (*P* = 0.019 and *P* = 0.0003) (Supplementary Fig. 1g–h). Beta diversity of gut microbiota between juvenile and adult in FWCGA were similar (*P* = 0.8) (Supplementary Fig. 1f), but it was significantly different between juvenile and adult in FOCGA (*P* = 0.001) (Supplementary Fig. 1i). These data suggest that environmental factors were critical to shape the gut microbiota. Moreover, the sex of feral swine had no significant impact on the formation of microbiota composition in this study (Supplementary Fig. 1j–l).

Microbiota of cattle was more similar to FWCGA than that of FOCGA measured by weighted UniFrac distances (Fig. 4d and Supplementary Fig. 2, and Supplementary Data 1, *P* < 0.0001). The relatedness of the microbiota communities analyzed by Unweighted Pair Group Method with Arithmetic mean (UPGMA) showed that feral swine microbiomes were clearly separated into two clusters based on sampling location, except a few samples that were interspersed with another sampling group. Microbiota communities of cattle were closer to FWCGA than that of FOCGA (Fig. 4e). Furthermore, based on our global positioning system (GPS) data of the animal space used for FWCGA and FOCGA, as shown in Fig. 4f, the home ranges of FOCGA were distributed mainly outside of cattle grazing area with no spatial overlap with cattle home ranges or their grazing pastures, while the home ranges of cattle were completely overlapped with FWCGA. We also observed that FOCGA and FWCGA had distinct home ranges with limited overlapped activity space, which may explain the few samples we found to be interspersed between FOCGA and FWCGA. Taken together, these results indicate that the dissimilarities of microbiome between FOCGA and FWCGA are consistent with the hypothesis that cohabitation within cattle grazing area is a critical factor for shaping microbiome structure of feral swine.

### Potential transmission of microorganisms at the wildlife-livestock interface

**Fig. 5:**
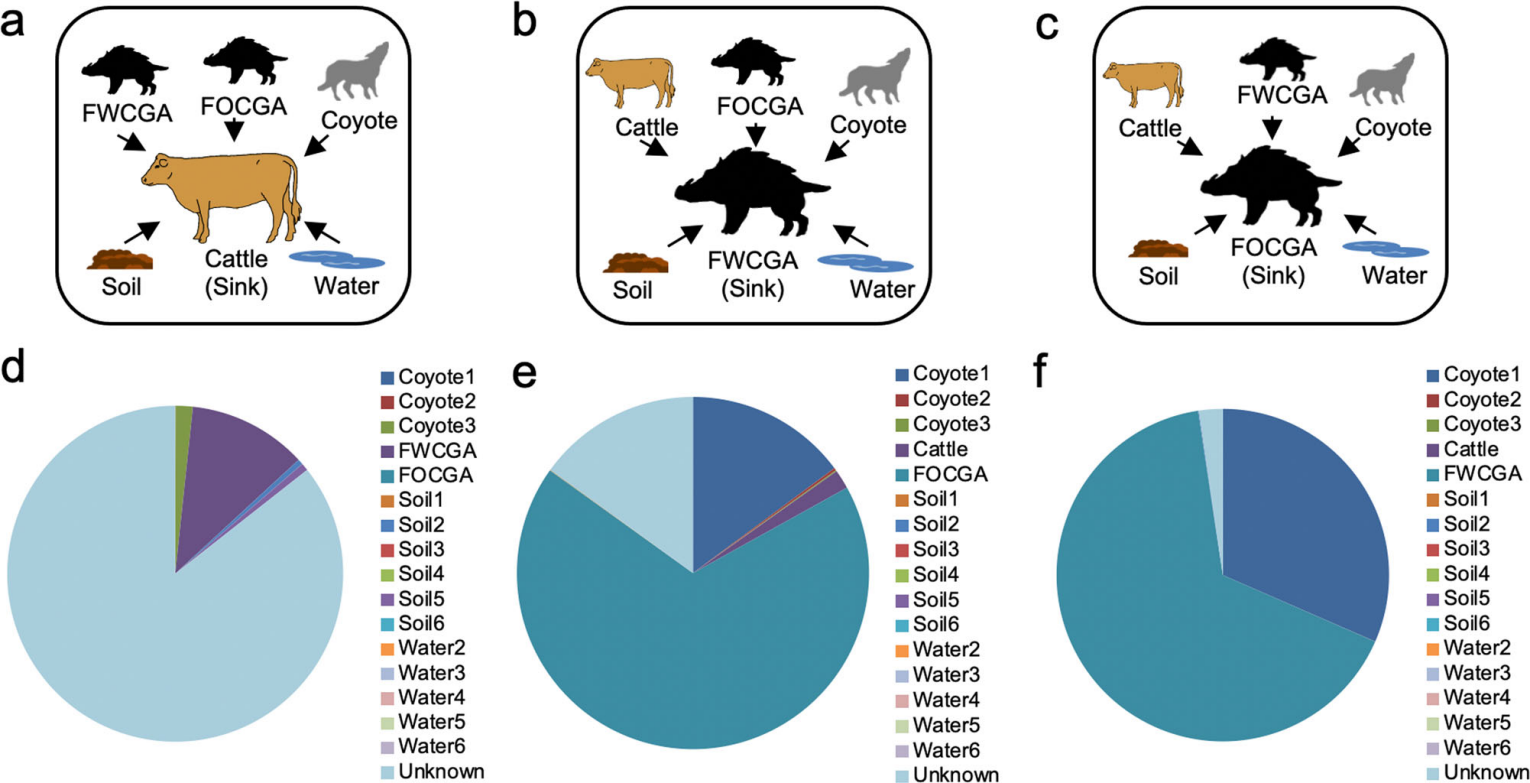
Source of bacteria between cattle and wildlife. Schematic diagram of SourceTracker analysis when cattle (**a**), FWCGA (**b**), and FOCGA (**c**) are the sinks, respectively. SourceTracker estimates microbiota transmission between sinks and source. Cattle (**d**), FWCGA (**e**), and FOCGA (**f**) are designated as a sink, respectively. Data from cattle, FWCGA, and FOCGA are the means of individual animals; each coyote, soil, and water samples were designated as a single source, respectively. Colors in the pie charts distinguish sources.

Since the microbiota community of cattle was more similar to FWCGA compared to FOCGA, we further investigated potential transmission of microorganisms among animal groups (cattle, FWCGA, and FOCGA). A transmission pathway analysis using SourceTracker was performed to determine the source of microbial communities in the sink samples (i.e., recipient). First, cattle microbiomes were designated as the sink samples and others were designated as sources (Fig. 5a). The majority (85.6%) of cattle microbiome was conserved among cattle. FWCGA contributed to cattle microbiome by ~11% (Fig. 5d and Supplementary Data 1). On the other hand, FOCGA had almost no influence on cattle (i.e., 0.003% of cattle microbiome originated from FOCGA). Other microbiomes including coyotes and environmental samples had little influence on cattle microbiota, only 3% combining all samples (Fig. 5d and Supplementary Fig. 3, and Supplementary Data 1). To understand the direction of bacteria transmission, we analyzed the opposite direction as well, i.e., considering FWCGA (Fig. 5b) and FOCGA (Fig. 5c) as a sink, respectively, and other microbiomes as sources. Approximately 66–68% of microbiome communities of FWCGA and FOCGA were shared (Fig. 5e, f, and Supplementary Fig. 4 and 5, and Supplementary Data 1). Compared to the contribution of FWCGA on cattle microbiome communities, cattle had only a minimal effect on feral swine. Only 2% and 0.003% of microbiota communities of FWCGA (Fig. 5e and Supplementary Fig. 4, and Supplementary Data 1) and FOCGA (Fig. 5f and Supplementary Fig. 5, and Supplementary Data 1) were predicted to be originated from cattle, respectively. These data indicate that cohabitation enhances transmission of microorganisms between animals.

### Identification of shared microorganisms between cattle and feral swine

**Fig. 6:**
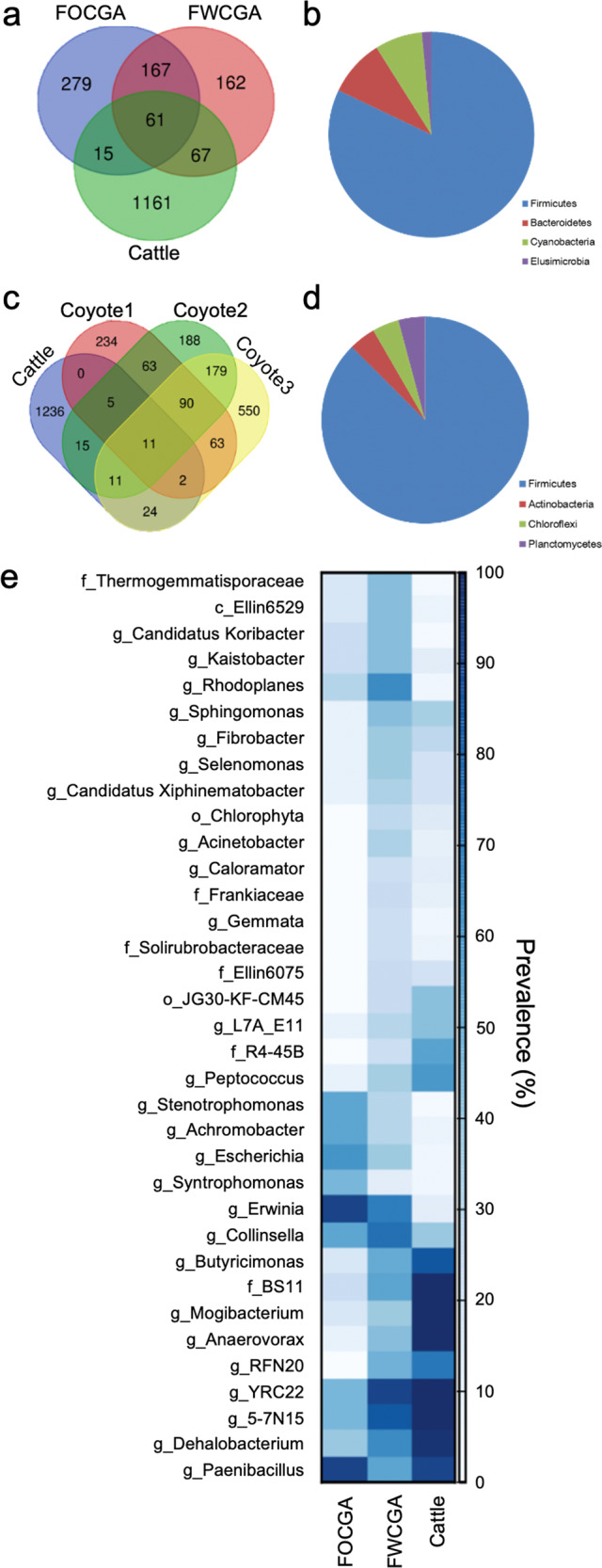
Shared bacteria between cattle and swine groups. **a** Co-occurrence of OTUs between cattle and swine groups (FWCGA and FOCGA). Core-OTUs from cattle and swine groups that were present in >50% of the samples were identified and compared among groups. **b** Bacterial classification of overlapped OTUs between FWCGA and cattle at the phylum level. **c** Co-occurrence of OTUs between cattle and coyotes. In coyote samples, OTUs in individual samples were used. **d** Bacterial classification of overlapped OTUs between coyote 3 and cattle at the phylum level. **e** The prevalence of core-bacteria in cattle, FWCGA, and FOCGA at the genus taxonomic level was compared. Only classified bacteria showing prevalence differences between FWCGA and FOCGA >20% were included.

To further identify co-occurring OTUs between cattle and wildlife samples, core-OTUs presenting in >50% of samples were identified in the microbiomes of cattle, FWCGA, and FOCGA, respectively. Besides 61 OTUs detected in all three microbiomes (i.e., cattle, FWCGA, and FOCGA), FWCGA contained 67 identical OTUs with cattle, while FOCGA had only 15 identical OTUs (Fig. 6a), indicating that FWCGA had more common bacteria with cattle than that of FOCGA. The 67 OTUs were comprised of Firmicutes (82%), Bacteroidetes (9%), Cyanobacteria (7.5%), and Elusimicrobia (1.5%) phyla with 11 genera, which were *[Prevotella]*, *Prevotella*, *Bacillus*, *Streptococcus*, *Clostridium*, *Coprococcus*, *Dorea*, *Faecalibacterium*, *Oscillospira*, *Ruminococcus*, and *Phascolarctobacterium* (Fig. 6b, Supplementary Data 1 and Supplementary Data 3). Coyote 3, trapped within the cattle grazing area, had 24 overlapping OTUs with cattle, which was the highest number in coyotes, except for core-OTUs among coyotes (Fig. 6c). The 24 OTUs were classified into Firmicutes (88%), Actinobacteria (4%), Chloroflexi (4%), and Planctomycetes (4%) phyla with six genera, which were *Adlercreutzia*, *SHD-231*, *Bacillus*, *Anaerostipes*, *Butyrivibrio*, and *Dorea* (Fig. 6d, Supplementary Data 1 and Supplementary Data 4).

We next compared the prevalence of bacteria to identify bacteria commonly presenting in FWCGA and cattle. From the identified core-bacteria presenting in >50% samples (Supplementary Data 5–7), we included classified bacteria with prevalence differences of >20% between FWCGA and FOCGA (Fig. 6e). 80% (28 out of 35) of bacteria showed similar prevalence between FWCGA and cattle, except the Ellin6529 class, the *Thermogemmatisporaceae* family, the *Candidatus Koribacter*, *Kaistobacter*, *Rhodoplanes*, *Collinsella* and *Paenibacillus* genera. Notably, ten bacterial taxa were only identified in FWCGA and cattle microbiota samples but were not isolated from FOCGA. Those taxa belong to JG30-KF-CM45 and Chlorophyta orders; *Ellin6075*, *Frankiaceae*, *Solirubrobacteraceae*, and *R4-45B* families; and *Caloramator*, *RFN20*, *Gemmata*, and *Acinetobacter* genera. Supplementary Data 8 lists the prevalence for all bacteria.

### High abundance of antibiotic resistance genes in feral swine

**Fig. 7:**
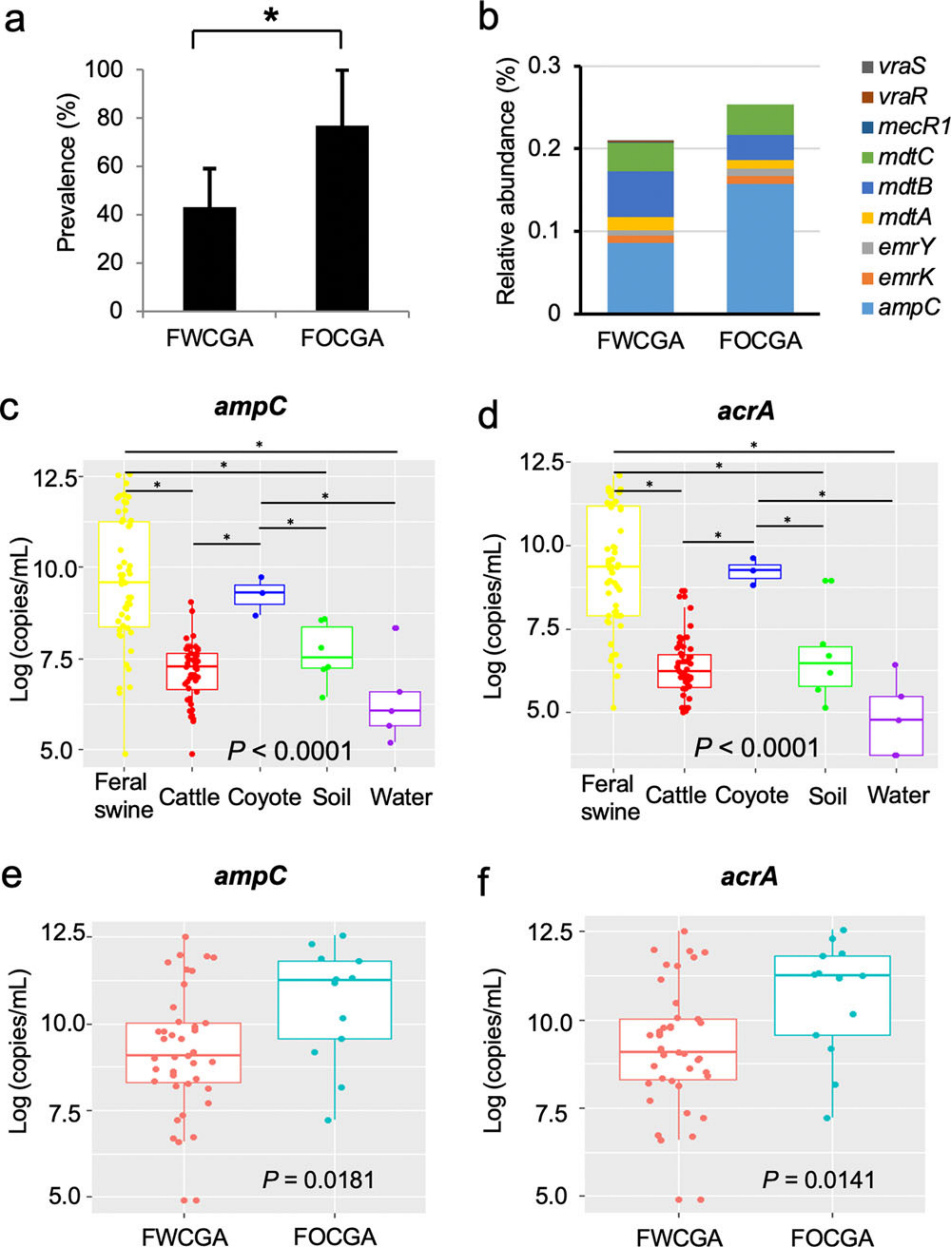
Proportion of antimicrobial resistance in FWCGA, FOCGA, and cattle. **a** The prevalence of CRBs in FWCGA and FOCGA. An asterisk indicates a statistical difference (*P* < 0.05) of each category comparison by Chi-Square test. **b** Relative abundance of antibiotic resistance genes in FWCGA and FOCGA. **c** Abundance of *ampC* gene in cattle, wildlife, and the environment. **d** Abundance of *acrA* gene in cattle, wildlife, and the environment. **e** Abundance of *ampC* gene in FWCGA and FOCGA. **f** Abundance of *acrA* gene in FWCGA and FOCGA. Box plots display the median (center line), 25th and 75th percentiles (box) and 5th and 95th percentiles (whiskers) for each individual. Asterisks indicate a statistical difference (*P* < 0.05) of each category comparison.

As transmission of microorganisms occurs at the wildlife-livestock interface (Fig. 5), and microbiota composition and structure of FWCGA and FOCGA were significantly different (Fig. 4), we hypothesized that the prevalence of ARMs, including CRB, was also different between the two feral swine groups. Consistent with our hypothesis, as shown in Fig. 7a, FOCGA showed a significantly higher prevalence of CRB than FWCGA (*P* = 0.03). Shotgun metagenomic sequencing analysis, which was conducted to investigate the difference in the proportion of ARGs harbored in gut microbiota of FWCGA and FOCGA groups, showed higher relative abundance of ARGs in FOCGA compared to FWCGA (Fig. 7b). ARGs encoding β-lactamase (*ampC*), multidrug resistance protein (*emrYK*), multidrug efflux system (*mdtCBA*), methicillin resistant protein (*mecR*1), and vancomycin resistance proteins (*vraRS*) were identified. To further validate whether FOCGA harbor more ARGs compared to FWCGA, qPCR was conducted to detect the abundance of the β-lactam resistance gene (*ampC*) and multi-drug resistance gene (*acrA*). Feral swine showed significantly higher abundance of *ampC* and *acrA* genes in their GI tract compared to cattle, soil, and water samples (Fig. 7c, d), which is consistent with the higher prevalence of ampicillin resistant bacteria in feral swine compared to cattle (Fig. 2a). Furthermore, *ampC* and *acrA* were significantly higher in FOCGA than FWCGA (Fig. 7e, f), which is consistent with the CRB prevalence data (Fig. 7a and Supplementary Data 1). Taken together, cattle and FWCGA had lower ARGs and CRB prevalence in the GI tract compared to FOCGA.

### Microbe-microbe interactions that affect the presence of CRB

**Fig. 8:**
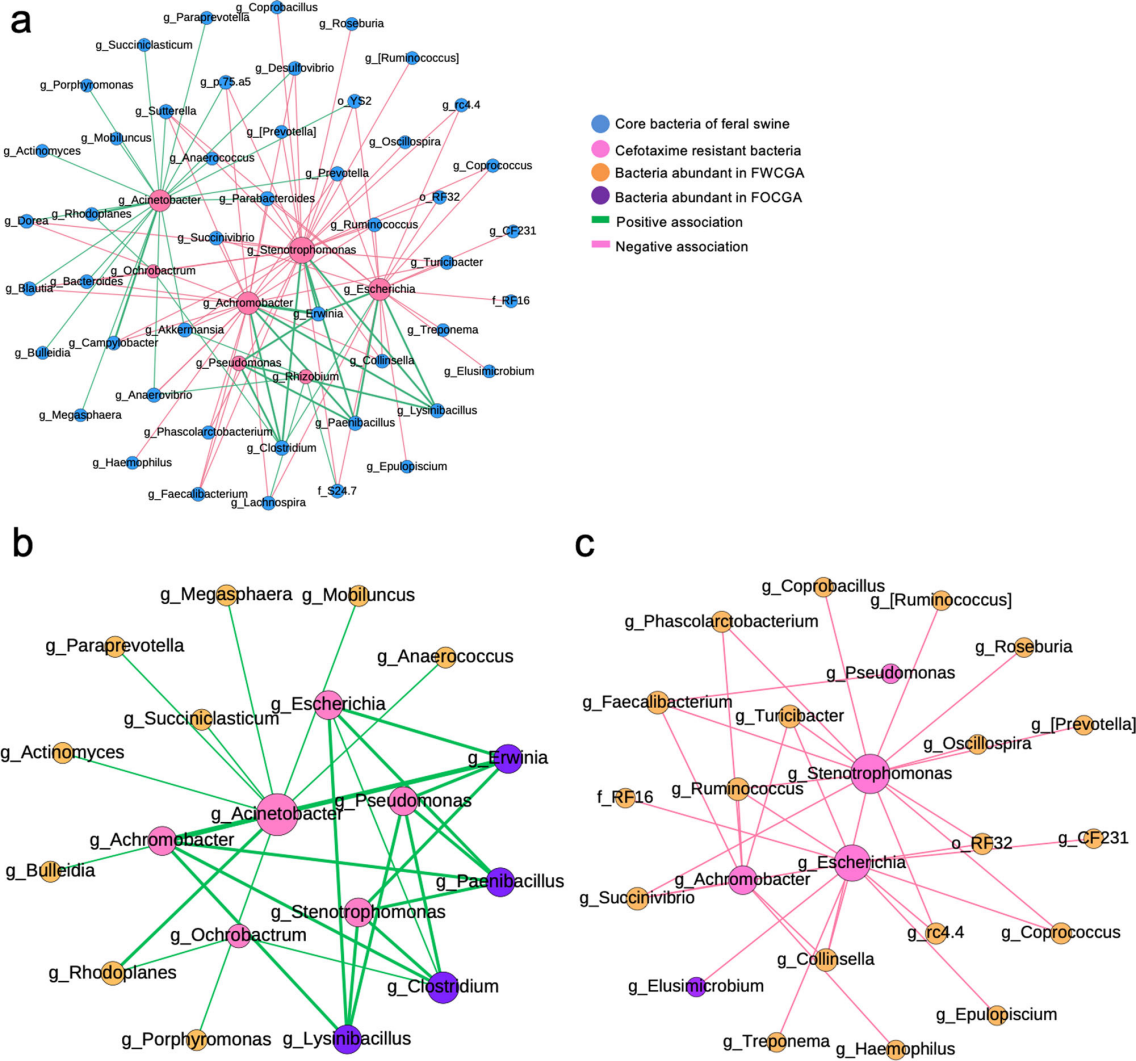
Association between core-bacteria and cefotaxime resistant bacteria (CRB) in feral swine. **a** Co-occurrence bacterial network between core-bacteria of feral swine and major CRB genera. **b** Network analysis showing positive associations between core-bacteria of feral swine and CRB. **c** Network analysis showing negative associations between core-bacteria of feral swine and CRB. Each pair of correlations has *P*-value < 0.05 and correlation coefficients above 0.2. Node size indicates the number of connections between taxa and thickness of the lines indicates the strength of relatedness.

Based on the prevalence of CRB and ARGs, we hypothesized that the gut microbiota of cattle and FWCGA may carry microorganisms that have a negative impact on CRB colonization in the GI tract. To identify bacteria-bacteria interactions, we conducted a co-occurrence network analysis between core-bacteria of feral swine and all CRB genera that were determined by full length 16 S rRNA gene sequencing shown in Fig. 2b (Supplementary Data 9–11). A total of 108 associations among 54 bacterial taxa (seven from CRB and 47 from core-bacteria) were identified (Fig. 8a, *P* < 0.05, r_s_ > 0.2 or r_s_ < −0.2). Forty-three associations (39.8%) were positively correlated, and 65 associations (60.2%) were negatively correlated (Fig. 8a). To understand the difference in the prevalence of CRB between two swine groups, we separated the networks based on taxa that had either positive (Fig. 8b) or negative (Fig. 8c) correlations with at least one genus of major CRB consistently. Thirteen bacterial taxa showed consistent positive correlations with CRB. Of these, nine bacteria were more abundant in FWCGA than FOCGA, whereas four taxa were more abundant in FOCGA (Fig. 8b). In particular, the four bacterial taxa, which were abundant in FOCGA (*Erwinia*, *Paenibacillus*, *Clostridium*, and *Lysinibacillus* genera), were strongly correlated with CRB presence compared to other bacterial taxa, which were abundant in FWCGA (Fig. 8b). Twenty bacteria represented negative correlations all the time (Fig. 8e). Interestingly, the relative abundance of all bacteria showing negative correlations with CRB was higher in FWCGA compared to FOCGA, except one genus (*Elusimicrobium*) (Fig. 8c). Through co-occurrence network analysis between feral swine commensal bacteria and CRB, we found that gut microbiota of FWCGA harbored more bacteria which have negative correlations with CRB. Taken together, the existence of those bacteria may inhibit the colonization of CRB, resulting in lower prevalence of CRB in FWCGA. Consistently, core bacteria of cattle also contained more bacteria with negative correlations to CRB (15/20) than bacterial taxa that were positively correlated with CRB (5/13), coinciding with low prevalence of CRB in cattle (Fig. 2a).

## Discussion

We found that ARMs against five medically important antibiotics are predominant at the wildlife-livestock interface. Especially striking finding was that the prevalence of cefotaxime-resistant bacteria was higher in wildlife and environmental samples than grazing cattle. More generally, we found that cohabitation of cattle and wildlife may affect the prevalence of antibiotic resistance and shapes microbiota structure at the wildlife-livestock interface.

We illustrated distinct bacterial composition and structure in the GI tract of livestock, wildlife, and the environment with significant similarities of microorganisms occurring among them. The pattern of more similar gut microbiota among individuals that spent more time in the same locations suggested transmission of gut microbiota between species. It is known that diet is the dominant factor in shaping the gut microbiota, even though gut microbial communities can be influenced by diverse intrinsic and extrinsic factors^25–27^. Cattle are herbivores while feral swine and coyotes are omnivores. Correspondingly, the gut microbiota of all animals clustered by diet type^28^. Although the diet of cattle and swine is generally different, cattle and swine that shared the same resources developed similar gut microbiota profiles. The fact that cattle showed higher bacterial diversity and richness than feral swine and coyotes is consistent with previous work showing that ruminants harbor more diverse microbiota than monogastric animals because of the metabolic potential and nutritional diversity of bacteria in ruminants^29,30^. However, when we investigated microbiota of feral swine after dividing samples into two groups based on the sampling location, we found that microbiota of two different species were more similar based on their proximity. Microbiota of FWCGA was closer to cattle compared to the similarity between FOCGA and cattle, and FWCGA had higher bacterial diversity and richness compared to that of FOCGA, suggesting that sharing the same environments contributed to shaping the microbiota of feral swine and cattle.

The source tracking analysis suggested that transmission of gut microbiota among feral swine and cattle may be occurring, especially for cattle using the same space as feral swine, emphasizing the risk of pathogen as well as ARMs transmission at the wildlife-livestock interface^17^. Although direct transmission of bacteria from feral swine to cattle was suggested^31^, it is unclear if cattle received microorganisms directly from feral swine in this study. Cattle might acquire bacteria from feral swine indirectly by digesting forage grasses that have been disturbed and contaminated by feral swine rooting behavior^32,33^. However, microbiota of FWCGA appeared unaffected by cattle microbiota, indicating the transmission of microorganisms may not be reciprocal. Alternatively, one explanation could be that the colonization of bacteria originating from monogastric feral swine is relatively high in ruminants’ digestive systems because ruminants have more complex digestive systems^34^. Thus, bacteria from feral swine might have increased opportunity to colonize in the gut of cattle relative to other species, while bacteria from cattle might have failed to colonize in the GI tract of feral swine. Further studies are necessary to understand how the colonization ability of bacteria is different in different hosts (i.e., ruminants or monogastrics), and how the frequency and length of feral swine’s visit in ranch affect the similarity between gut microbiota profiles among cattle and feral swine (i.e., exposure risk).

Gut microbiota structure affects the level of colonization resistance against pathogenic bacteria due to competition for resources^35^. For example, immature microbiota, such as in infants, or microbiota disturbance through antibiotic treatment is more vulnerable to pathogens, indicating that gut microbiota have a critical role in preventing colonization of pathogens and ARMs^36,37^. Our results indicated that FOCGA showed a higher prevalence of CRB, and the prevalence of individual bacteria showed a difference (>20%) between FOCGA and FWCGA (Fig. 6e). Notably, the prevalence of those bacteria between FWCGA and cattle presented similar patterns, suggesting that the higher or lower prevalence of bacteria listed in Fig. 6e might be associated with lower prevalence of CRB. Furthermore, co-occurrence network analysis showed that microbe-microbe interactions might have prevented colonization of specific bacteria including CRB (Fig. 8). Even though diets of cattle and feral swine are different, they shared similar gut microbiota with similar network profiles. We found 20 bacterial taxa in feral swine showing negative correlations with the major CRB genera (RF32 order, *RF16* family, *[Prevotella]*, *[Ruminococcus]*, *CF231*, *Collinsella*, *Coprobacillus*, *Coprococcus*, *Elusimicrobium*, *Epulopiscium*, *Faecalibacterium*, *Haemophilus*, *Oscillospira*, *Phascolarctobacterium*, *rc4.4*, *Roseburia*, *Ruminococcus*, *Succinivibrio*, *Treponema*, and *Turicibacter* genera) (Fig. 8c). Of these, 19 bacteria (except *Elusimicrobium* genus) were more abundant in FWCGA than FOCGA, and cattle also contained those bacteria except *Collinsella*, *Haemophilus*, *[Ruminococcus]*, and *Succinivibrio*. These data suggest that the presence of the 15 bacteria may inhibit colonization of CRB in FWCGA and cattle. Consistent with our findings, extended-spectrum β-lactamases (ESBLs) producing bacteria in humans was decolonized through fecal microbiota transplantation by improving gut microbiota in patients^38^. In particular, when fecal materials with a higher abundance of *Barnesiella*, *Bacteroides*, and *Butyricimonas* were transplanted to patients with New Delhi Metallo-β-lactamase (NDM)-producing *Klebsiella pneumonia*, NDM-producing *K. pneumonia* were decolonized from 57% of the patients (4/7)^38^. Piewngam et al. also found that ESBL-producing *Enterobacteriaceae* carriers had a significantly higher abundance of Firmicutes, while non-carriers had a higher abundance of Bacteroidetes^39^.

In order to understand how ARMs, transmit at the wildlife-livestock interface, we investigated antibiotic resistance of five medically important antibiotics to identify potential transmission of ARMs. We found that feral swine had higher prevalence of ARMs against ampicillin, cefotaxime, and tetracycline in the GI tract that was significantly higher compared to cattle. Although wild animals were not intentionally given antibiotics, they could present a higher prevalence of ARMs as a potential reservoir. Feral swine has diverse habitats and can acquire ARMs directly or indirectly through the shared environments with human and livestock (i.e., pasture, water, and soil)^40^. Interestingly, bacterial richness and diversity in FOCGA were significantly lower than FWCGA (Fig. 4), suggesting that microbiota composition may play a role in the prevalence of ARMs and ARGs. This observation is supported by the findings that healthy gut microbiota is negatively associated with the colonization of ARMs^41^. In addition, feral swine living closer to cattle showed lower CRB prevalence than the feral swine that were located further away (Fig. 7a and Supplementary Data 1). The high prevalence of CRB in feral swine compared to cattle suggests potential transmission of ARMs from feral swine to cattle. These data suggest that there is a substantial risk of cattle acquiring CRB from feral swine.

There may be a limitation in the number of samples included in this study, especially unbalanced sample sizes between FWCGA and FOCGA and low number of samples from coyote (*n* = 3), especially due to difficulties to trap wild animals. Future studies are needed to add more samples and data collection points to determine whether our findings are reliable. In addition, it is necessary to investigate other wildlife reported to interact with cattle, such as birds, deer, elk, and badgers as an extrinsic factor in shaping the microbiota of cattle^42–45^.

The population of feral swine in the U.S. has been expanding rapidly over the last several decades^46^ despite substantial control efforts^47^. There are currently about six million feral swine in 35 states including Florida^48^, causing increased damage to agricultural crops, natural resources, endangered species, and private property^49,50^. Furthermore, their contact ecology may support transmission of zoonotic and agricultural diseases such as rabies, bovine tuberculosis, classical swine fever, and brucellosis, showing the importance of feral swine control^20,32,51,52^. Our results support the hypothesis that feral swine may readily transmit microorganisms to cattle by showing that the closer feral swine are to cattle, the more likely they are to share the same microbiota. Feral swine could interact with cattle both indirectly and directly, however, indirect contact rate is usually higher than direct contact rate^53^. As indirect contacts are most common at food and water sites^54,55^, environments could be vehicles for transferring bacteria between wildlife and livestock.

## Methods

### Ethics statement

Research protocols for animal capture, animal care, and animal use were approved by the University of Florida [Protocols for feral swine (#201408495 & #201808495) for coyote (#201408477), and for cattle (#201709994)]. All the animals were handled and immobilized following approved standard practices and either released back into the wild after capture, or for cattle sampled during standard chute side ranch management practices.

### Sample collection and isolation of antibiotic-resistant microorganisms

We studied cattle and wildlife at Buck Island Ranch in South Florida (Fig. 1). Animal feces were collected in April and May 2017 from the recto-anal junction of cattle (*Bos taurus*; *n* = 47), feral swine (*Sus scrofa*; *n* = 52), and coyotes (*Canis latrans*; *n* = 3) using sterile cotton swabs. All cattle were pasture-grazing females between 3 and 8 years of age. Fecal samples from feral swine were collected from 10 juvenile (3–12 months) and 42 adults (≥12 months). Environmental samples were obtained within the cattle ranch including soil (*n* = 6) and water (*n* = 5) samples. Swab samples from animals were resuspended with 2 ml of TSB and 2 ml of 30% glycerol. Soil samples were weighed first then suspended with 30% glycerol. In the case of water samples, bacterial cells were collected by centrifugation at 3400 × g for 10 min and the pellet was resuspended with 1 ml of TSB and 1 ml of 30% glycerol. All processed samples were stored at −80 °C until use. To isolate antibiotic-resistant microorganisms (ARMs), processed sample solutions were spread on MacConkey agar plates (BD, USA) containing ampicillin (50 μg/mL), cefotaxime (4 μg/mL), kanamycin (50 μg/mL), streptomycin (100 μg/mL), or tetracycline (15 μg/mL)^4^, and the plates were incubated overnight at 37 °C. For the bacterial identification, genomic DNA was extracted from randomly selected cefotaxime resistant bacteria (CRB) with a bead-beating method, and 16 S rRNA gene was amplified as described previously^56^. 16 S rRNA amplicons were sequenced after PCR purification. The resulting sequences were compared against the NCBI database to identify bacterial species and a maximum likelihood phylogeny was constructed by MEGAX based on the Jukes and Cantor model with 1000 bootstrap replications^57^.

### Movement data and space use for cattle and feral swine

To explicitly understand the space use of the feral swine and cattle involved in this study, we deployed global positioning system (GPS) collars on cattle and swine during the captures and programed the collars to record GPS fixes with a 30-min interval from April to May in 2017. Animal space use is often quantified by the utilization distributions (UDs; i.e., the intensity of space use for an animal)^58^. Home range delineates the frequently used areas by animals for their daily activities^59^. Here, we estimated the UDs for each feral swine and cattle using kernel density estimation with the least-squares cross-validation bandwidth using “adehabitatHR” R-package^60^. The 95% isopleth of UDs was used to define the home range of each animal. We then merged the home ranges for different groups [cattle, feral swine caught within cattle grazing areas (FWCGA), and feral swine caught outside cattle grazing areas (FOCGA)], to visualize their spatial overlap.

### 16S rRNA gene sequencing

One of the sterile cryotube vials (2 ml) containing suspended sample solutions was centrifuged to collect a pellet. The pellet was used to extract genomic DNA with QIAamp PowerFecal DNA kit (Qiagen, USA) under the manufacturer’s instruction. A polymerase chain reaction (PCR) was performed to amplify V4 region of 16 S rRNA gene with dual-index primers as previously reported^61^. The PCR amplicons were purified and normalized using SequalPrep Normalization plate kit (Invitrogen, USA) and the DNA concentration was measured with Qubit 3.0 Fluorometer (Invitrogen, USA). The same amount of DNA was pooled from each sample to construct a DNA library. Quality of the pooled DNA library was determined using the Agilent 2200 TapeStation System and qPCR to ascertain the functionality of the library and the final DNA library was sequenced with MiSeq v2, 2 × 250 cycle cartridge (Illumina, USA) on an Illumina Miseq platform at Interdisciplinary Center for Biotechnology Research (ICBR) at University of Florida.

### Microbial community analysis

Raw 16 S rRNA sequencing data were analyzed using the QIIME pipeline (1.9.0)^62^. Raw sequencing reads were demultiplexed and assembled to paired-end reads with multiple_join_paired_ends.py and multiple_split_libraries_fastq.py scripts in QIIME. Chimeric sequences were identified with a usearch61 method, and the identified chimeric sequences were eliminated from a FASTA file using identify_chimeric_seqs.py and filter_fasta.py scripts. The filtered sequences were used to cluster Operational Taxonomic Units (OTUs) and assigned to the taxonomical levels following the open-reference workflow against the SILVA database (https://www.arb-silva.de/documentation/release-132/) with 99% identity. Final OTU table were rarefied to a lowest sequencing depth among samples, which is 21,216, and two samples that had a smaller number of reads than 21,216 were excluded from further analysis. Alpha (Chao1 and Shannon index) and beta diversity (weighted UniFrac distances) indices were determined with alpha_diversity.py and core_diversity_analyses.py scripts, respectively. To calculate genetic distances between cattle and wildlife samples, weighted UniFrac distances were compared among samples. SourceTracker software installed in QIIME was used to determine the extent of shared microbiota between cattle, wildlife, and environmental samples. SourceTracker is a Bayesian approach to estimate the proportional contributions of bacterial taxa from potential sources to a given sink simultaneously^63^. This method can determine the proportion of OTUs with 99% identity from a given set of sources that contribute to the sink. OTUs that existed in less than two samples were excluded from the OTU table using filter_otus_from_otu_table.py script, and the filtered OTU table was converted from the BIOM format to the tab-separated text format using QIIME. In the mapping file, cattle, FWCGA, and FOCGA were designated as sink and source, respectively, when running SourceTracker^63^.

### Quantitative PCR for antibiotic resistance genes

Real-time quantitative PCR (qPCR) was conducted to quantify *ampC* and *acrA* genes in DNA extracted from all samples described above. The qPCR assays were performed using the SsoAdvanced Universal SYBR Green Supermix (Bio-Rad, USA) on the CFX96 Touch™ Real-Time PCR Detection System (Bio-Rad, USA). The primer sets used for detecting the *ampC* gene are KCP947 (5'-CCTCTTGCTCCACATTTGCT-3') and KCP948 (5'-ACAACGTTTGCTGTGTGACG-3'), and for *acrA* gene are KCP945 (5'-CTCTCAGGCAGCTTAGCCCTAA-3') and KCP946 (5'-TGCAGAGGTTCAGTTTTGACTGTT-3'). The real-time qPCR program was as follows: initial denaturing at 95 °C for 5 min, followed by 40 cycles of 10 s at 95 °C, 30 s at different annealing temperatures (58 °C for *ampC* gene, 60 °C for *acrA* gene), and 30 s at 72 °C. The fluorescence data were acquired at 72 °C, and the final melting curve was constructed with temperature ramping up from 65 to 95 °C. Standard curves were prepared using the genomic DNA of *E. coli* JEONG9592^6^. A five-fold serial diluted calibration curve for each gene was tested in triplicate on the same PCR plate.

### Shotgun metagenomic sequencing and downstream analysis

Extracted DNA aliquots of fecal samples from cattle, FWCGA and FOCGA were pooled together, respectively. The library for the three DNA pools was prepared according to the Nextera XT protocol, and the shotgun metagenomic sequencing was performed using the MiSeq Reagent Kit v2 through the Illumina MiSeq platform. Raw data files from shotgun sequencing were uploaded to the MG-RAST server. Pair-end sequences were merged and analyzed using MG-RAST pipeline. Briefly, artificial replicate sequences produced by sequencing artifacts and host specific species sequences (*Bos taurus* UMD v3.0 or *Sus scrofa*, NCBI v10. 2) were removed. Sequences with their length <50 bases and quality score <20 were trimmed for further analysis. Annotation was conducted using the KEGG orthology (KO), with a maximum e-value of 1×10^−5^ and a minimum identity cutoff and alignment length of 60% and 15 bp, respectively. The sequence counts were normalized to 48,382.

### Co-occurrence network analysis

To predict bacteria-bacteria interactions in gut microbiota, co-occurrence network between core-bacteria (presence in >50% of the samples) and major genus of CRB (identified in this study shown in Fig. 2) were tested using pairwise Spearman correlations based on the relative abundance of bacteria^64,65^. The Spearman correlations matrix was analyzed using Hmisc 3.9-3 package within the R software (version 3.6.1). The cutoff for each co-occurring pair was above a Spearman correlation coefficient of 0.2 with *P*-values under 0.05. The co-occurring networks were visualized with Gephi (http://gephi.github.io/). Nodes in the network indicate bacterial taxa and edges that connect two nodes represent significant correlations between bacteria. The size of nodes represents the number of connections among the nodes and the thickness of edges reveals the strength of correlation.

### Statistics and reproducibility

For the comparison of the ARMs prevalence among groups, a Chi-Square test was applied, and a one-way analysis of variance (ANOVA) test was applied followed by a post-hoc Tukey’s HSD test for evaluating the multiple comparisons of alpha diversity and qPCR data using GraphPad Prism (version 6) or R (version 3.6.1) software. An analysis of similarities (ANOSIM) was used to compare significant differences among microbiota structures of cattle and wildlife based on weighted UniFrac distances, and a parametric two-sided Student’s *t-*test was applied to compare the weighted UniFrac distances between FWCGA (*n* = 37) and FOCGA groups (*n* = 13) against cattle. The relative abundance of bacteria among different groups was compared with a group_significance.py script in QIIME by applying an ANOVA test. *P*-values < 0.05 were considered as statistically significant.

### Reporting summary

Further information on research design is available in the Nature Research Reporting Summary linked to this article.

## Supplementary information

Supplementary Information

Description of Additional Supplementary Files

Supplementary data1

Supplementary data2

Supplementary data3

Supplementary data4

Supplementary data5

Supplementary data6

Supplementary data7

Supplementary data8

Supplementary data9

Supplementary data10

Supplementary data11

Reporting Summary

## Data Availability

The 16 S metagenomics data generated and analyzed in the current study are available in the NCBI under the BioProject number PRJNA589650. Source data underlying main figures are presented in Supplementary Data 1.

## Code availability

All custom code is available from the corresponding author upon reasonable request.
