# Supplementary material for: Transmission of antibiotic resistance at the wildlife-livestock interface
Source: Commun Biol. 2022 Jun 15;5:585. doi: 10.1038/s42003-022-03520-8 (PMC9200806; doi:10.1038/s42003-022-03520-8)
Supplement: Supplementary file 1 — Supplementary Information [file 42003_2022_3520_MOESM1_ESM.pdf]

Supplementary information for

Transmission of Antibiotic Resistance at the Wildlife-Livestock Interface

Shinyoung Lee[1,2], Peixin Fan[1,2], Ting Liu[1,2], Anni Yang[3,4], Raoul K. Boughton[5], Kim M. Pepin[4], Ryan S. Miller[6], and Kwangcheol Casey Jeong[1,2*]

[1]Emerging Pathogens Institute, University of Florida, Gainesville, FL 32611, USA

[2]Department of Animal Sciences, University of Florida, Gainesville, FL 32611, USA

[3]Department of Fish, Wildlife, and Conservation Biology, Colorado State University, Fort Collins, CO 80523, USA

[4]National Wildlife Research Center, United States Department of Agriculture, Animal and Plant Health Inspection Service, Wildlife Services, 4101 Laporte Ave., Fort Collins, CO 80521, USA

[5]Range Cattle Research and Education Center, Wildlife Ecology and Conservation, University of Florida, Ona, FL 33865, USA

[6]Center for Epidemiology and Animal Health, United States Department of Agriculture, Animal and Plant Health Inspection Service, Veterinary Services, 2150 Center Dr., Fort Collins, CO 80523, USA

[*]**Corresponding author:**

K. Casey Jeong, PhD

Emerging Pathogens Institute and Department of Animal Sciences, University of Florida

Address: 2055 Mowry Rd, Gainesville, FL USA 32611

Email:kcjeong@ufl.edu, Phone: 1-352-294-5376

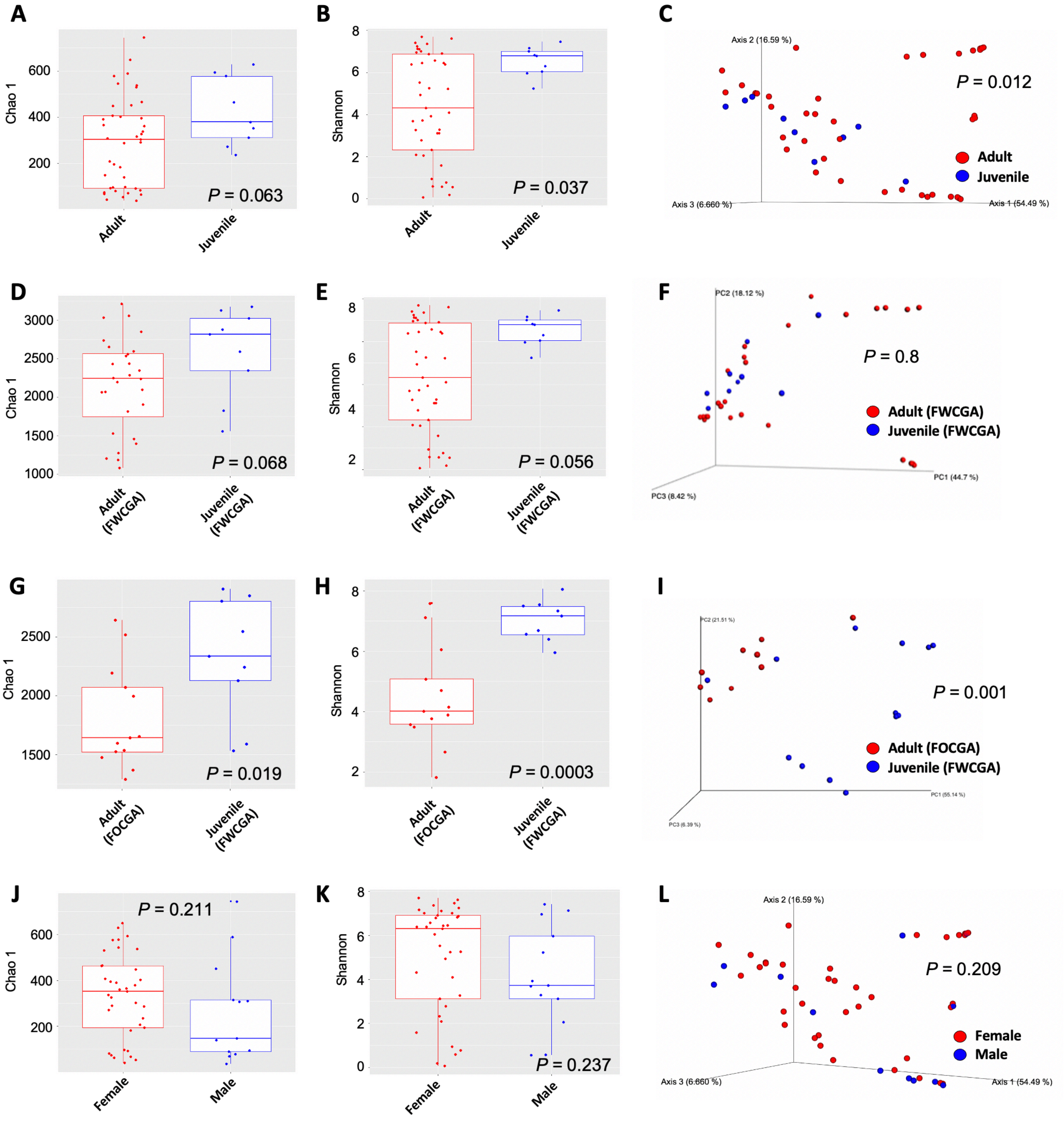

Supplementary Figure 1. The effects of age and sex on microbiota composition of feral swine.

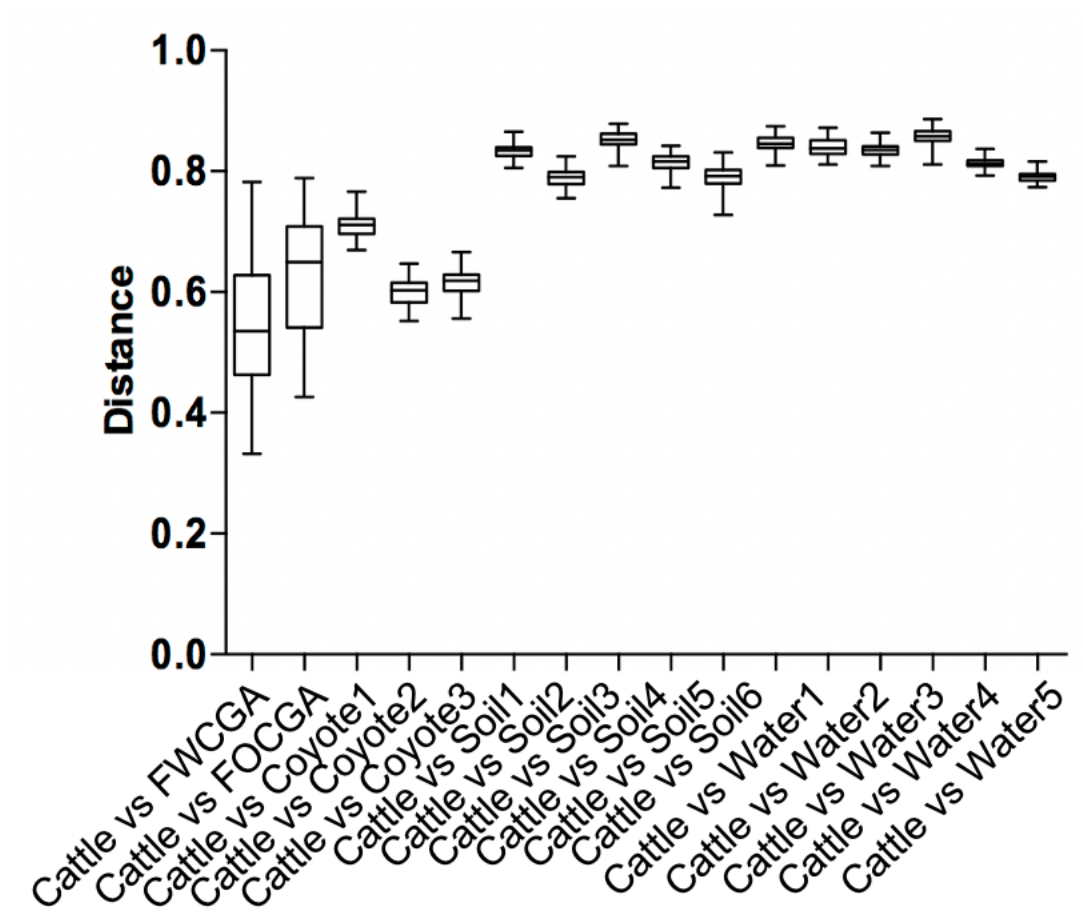

Supplementary Figure 2. Comparison of the weighted UniFrac distances between cattle and individual samples.

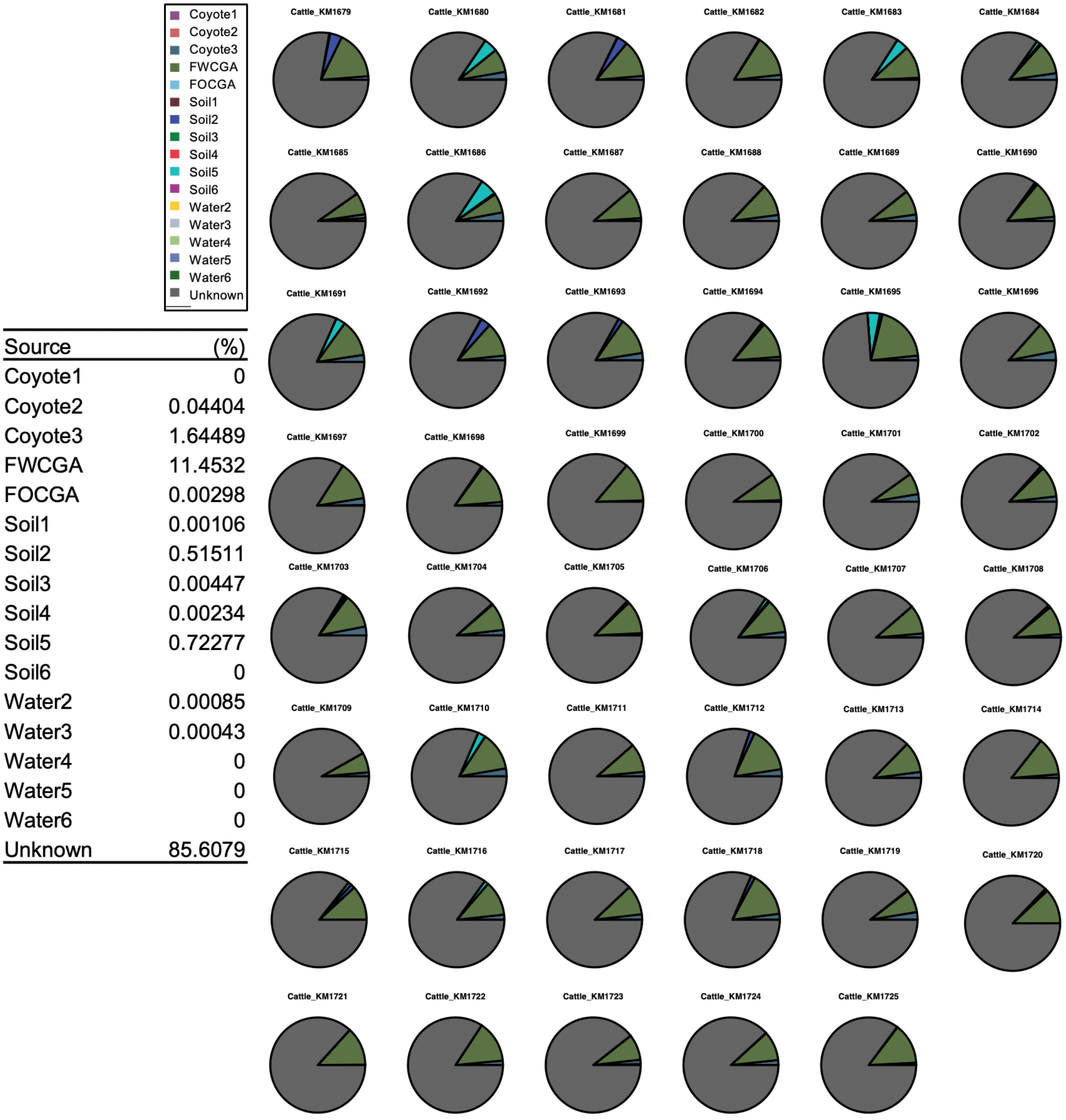

Source	(%)
Coyote1	0
Coyote2	0.04404
Coyote3	1.64489
FWCGA	11.4532
FOCGA	0.00298
Soil1	0.00106
Soil2	0.51511
Soil3	0.00447
Soil4	0.00234
Soil5	0.72277
Soil6	0
Water2	0.00085
Water3	0.00043
Water4	0
Water5	0
Water6	0
Unknown	85.6079

Supplementary Figure 3. SourceTraker analysis with cattle as a sink and other samples as sources.

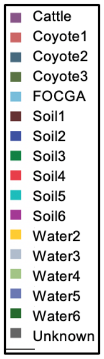

Source	(%)
Coyote1	14.84405
Coyote2	0.234595
Coyote3	0.106757
Cattle	1.754865
FOCGA	67.9673
Soil1	0.021892
Soil2	0.004865
Soil3	0.001892
Soil4	0.035676
Soil5	0.021351
Soil6	0.00027
Water2	0
Water3	0
Water4	0.00027
Water5	0
Water6	0.00027
Unknown	15.00595

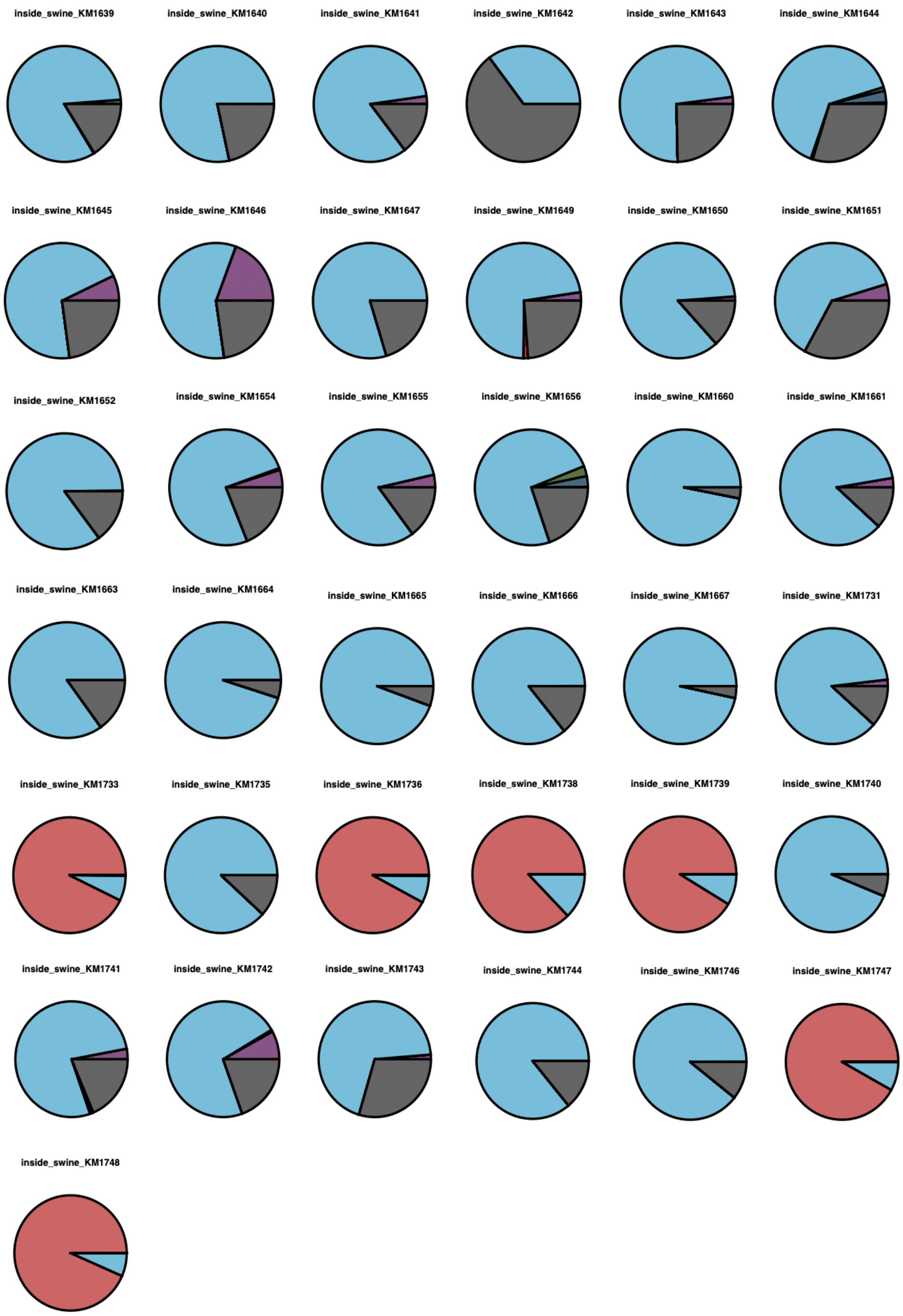

Supplementary Figure 4. SourceTraker analysis with FWCGA as a sink and other samples as sources.

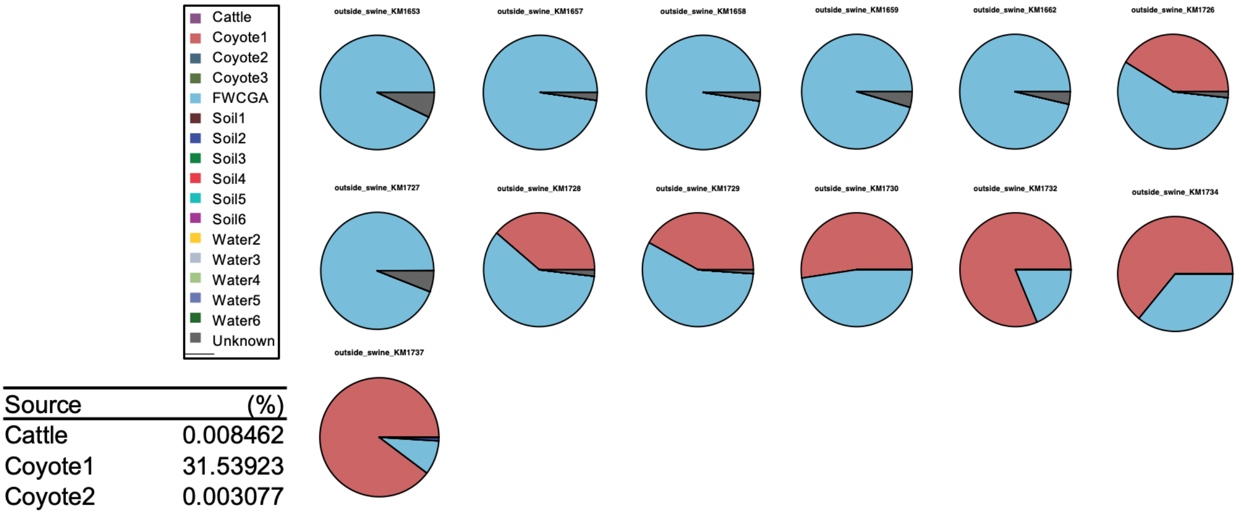

Source	(%)
Cattle	0.008462
Coyote1	31.53923
Coyote2	0.003077
Coyote3	0
FWCGA	66.06
Soil1	0
Soil2	0.070769
Soil3	0
Soil4	0
Soil5	0.005385
Soil6	0
Water2	0.000769
Water3	0
Water4	0.000769
Water5	0
Water6	0
Unknown	2.311538

Supplementary Figure 5. SourceTraker analysis with FOCGA as a sink and other samples as sources.
