# Supplementary material for: Transmission of antibiotic resistance at the wildlife-livestock interface
Source: Commun Biol. 2022 Jun 15;5:585. doi: 10.1038/s42003-022-03520-8 (PMC9200806; doi:10.1038/s42003-022-03520-8)
Supplement: Supplementary file 2 — Description of Additional Supplementary Files [file 42003_2022_3520_MOESM2_ESM.pdf]

Description of Additional Supplementary Files

File name: Supplementary Data 1
Description: The source data behind the Fig 2a, 3a-b, 3d, 4a-b, 4d, 5d-f, 6b, 6d, and 7a in the paper

File name: Supplementary Data 2
Description: Summary of the metagenomic sequencing in each sample

File name: Supplementary Data 3
Description: The source data of taxonomic classification of the overlapping OTUs between cattle and FWCGA

File name: Supplementary Data 4
Description: The source data of taxonomic classification of the overlapping OTUs between cattle and coyote caught within cattle grazing area

File name: Supplementary Data 5
Description: The source data of relative abundance of core-bacteria (≥50%) in cattle

File name: Supplementary Data 6
Description: The source data of relative abundance of core-bacteria (≥50%) in FWCGA

File name: Supplementary Data 7
Description: The source data of relative abundance of core-bacteria (≥50%) in FOCGA

File name: Supplementary Data 8
Description: The source data of comparison of bacteria prevalence among cattle, FWCGA, and FOCGA

File name: Supplementary Data 9
Description: The source data of relative abundance of the major CRB and corebacteria of feral swine in cattle

File name: Supplementary Data 10
Description: The source data of relative abundance of the major CRB and corebacteria of feral swine in FWCGA

File name: Supplementary Data 11
Description: The source data of relative abundance of the major CRB and corebacteria of feral swine in FOCGA
