# Supplementary material for: Transmission of antibiotic resistance at the wildlife-livestock interface
Source: Commun Biol. 2022 Jun 15;5:585. doi: 10.1038/s42003-022-03520-8 (PMC9200806; doi:10.1038/s42003-022-03520-8)
Supplement: Supplementary file 14 — Reporting Summary [file 42003_2022_3520_MOESM14_ESM.pdf]

nature portfolio

Corresponding author(s): Kwangcheol Casey Jeong

Last updated by author(s): Apr 28, 2022

Reporting Summary

Nature Portfolio wishes to improve the reproducibility of the work that we publish. This form provides structure for consistency and transparency in reporting. For further information on Nature Portfolio policies, see our Editorial Policies and the Editorial Policy Checklist.

Statistics

For all statistical analyses, confirm that the following items are present in the figure legend, table legend, main text, or Methods section.

n/a	Confirmed	
☐	☒	The exact sample size (n) for each experimental group/condition, given as a discrete number and unit of measurement
☐	☒	A statement on whether measurements were taken from distinct samples or whether the same sample was measured repeatedly
☐	☒	The statistical test(s) used AND whether they are one- or two-sided<br>*Only common tests should be described solely by name; describe more complex techniques in the Methods section.*
☒	☐	A description of all covariates tested
☒	☐	A description of any assumptions or corrections, such as tests of normality and adjustment for multiple comparisons
☒	☐	A full description of the statistical parameters including central tendency (e.g. means) or other basic estimates (e.g. regression coefficient) AND variation (e.g. standard deviation) or associated estimates of uncertainty (e.g. confidence intervals)
☐	☒	For null hypothesis testing, the test statistic (e.g. F, t, r) with confidence intervals, effect sizes, degrees of freedom and P value noted<br>*Give P values as exact values whenever suitable.*
☒	☐	For Bayesian analysis, information on the choice of priors and Markov chain Monte Carlo settings
☒	☐	For hierarchical and complex designs, identification of the appropriate level for tests and full reporting of outcomes
☒	☐	Estimates of effect sizes (e.g. Cohen's d, Pearson's r), indicating how they were calculated

Our web collection on statistics for biologists contains articles on many of the points above.

Software and code

Policy information about availability of computer code

Data collection	No software was used to collect the data in our study
Data analysis	GraphPad Prism (version 6)<br>R (version 3.6.1)<br>QIIME pipeline (1.9.0)<br>MG-RAST pipeline<br>Gephi

For manuscripts utilizing custom algorithms or software that are central to the research but not yet described in published literature, software must be made available to editors and reviewers. We strongly encourage code deposition in a community repository (e.g. GitHub). See the Nature Portfolio guidelines for submitting code & software for further information.

Data

Policy information about availability of data

All manuscripts must include a data availability statement. This statement should provide the following information, where applicable:

- Accession codes, unique identifiers, or web links for publicly available datasets
- A description of any restrictions on data availability
- For clinical datasets or third party data, please ensure that the statement adheres to our policy

All data are available in the paper or Supplementary Information.
Data and code availability. The 16S metagenomics data generated and analyzed in the current study are available in the NCBI under the BioProject number

PRJNA589650.
Any additional data can be requested from the corresponding authors.

Field-specific reporting

Please select the one below that is the best fit for your research. If you are not sure, read the appropriate sections before making your selection.

☐ Life sciences ☐ Behavioural & social sciences ☒ Ecological, evolutionary & environmental sciences

For a reference copy of the document with all sections, see nature.com/documents/nr-reporting-summary-flat.pdf

Ecological, evolutionary & environmental sciences study design

All studies must disclose on these points even when the disclosure is negative.

Study description	This is a report describing how antibiotic resistant bacteria transmit at the wildlife-livestock interface. By investigating the gut microbiome and antibiotic resistance profiles of feral swine, coyotes, and cattle, as well as environmental samples, we found ARMs transmission pathways between grazing livestock and wildlife.
Research sample	We studied cattle and wildlife at Buck Island Ranch in South Florida. Animal feces were collected from the recto-anal junction of cattle (n=47), feral swine (n=52), and coyotes (n=3). Environmental samples were obtained within the cattle ranch including soil (n=6) and water (n=5) samples.
Sampling strategy	No sample size calculation was performed. We caught as much as wildlife as we can
Data collection	*Describe the data collection procedure, including who recorded the data and how.*
Timing and spatial scale	All sample were collected during April, 2017 to May, 2017.
Data exclusions	No data were exclude from the analyses
Reproducibility	*Describe the measures taken to verify the reproducibility of experimental findings. For each experiment, note whether any attempts to repeat the experiment failed OR state that all attempts to repeat the experiment were successful.*
Randomization	*Describe how samples/organisms/participants were allocated into groups. If allocation was not random, describe how covariates were controlled. If this is not relevant to your study, explain why.*
Blinding	*Describe the extent of blinding used during data acquisition and analysis. If blinding was not possible, describe why OR explain why blinding was not relevant to your study.*

Did the study involve field work? ☐ Yes ☒ No

Reporting for specific materials, systems and methods

We require information from authors about some types of materials, experimental systems and methods used in many studies. Here, indicate whether each material, system or method listed is relevant to your study. If you are not sure if a list item applies to your research, read the appropriate section before selecting a response.

Materials & experimental systems

n/a	Involved in the study	
☒	☐	Antibodies
☒	☐	Eukaryotic cell lines
☒	☐	Palaeontology and archaeology
☐	☒	Animals and other organisms
☒	☐	Human research participants
☒	☐	Clinical data
☒	☐	Dual use research of concern

Methods

n/a	Involved in the study	
☒	☐	ChIP-seq
☒	☐	Flow cytometry
☒	☐	MRI-based neuroimaging

Animals and other organisms

Policy information about studies involving animals; ARRIVE guidelines recommended for reporting animal research

Laboratory animals	The study did not involve laboratory animal.
Wild animals	All cattle were pasture-grazing females between 3 and 8 years of age. Fecal samples from feral swine were collected from 10 juvenile (3-12 months) and 42 adults (≥12 months).

Field-collected samples	The study did not involve samples collected from the field.
Ethics oversight	Research protocols for animal capture, animal care, and animal use were approved by the University of Florida [Protocols for feral swine (#201408495 & #201808495) for coyote 372 (#201408477), and for cattle (#201709994)]. All of the animals were handled and immobilized following approved standard practices and either released back into the wild after capture, or for cattle sampled during standard chute side ranch management practices.

Note that full information on the approval of the study protocol must also be provided in the manuscript.
